# Perspectives on Bioengineering Clinical Immersion: History, Innovation, and Impact

**DOI:** 10.1007/s10439-020-02508-x

**Published:** 2020-04-20

**Authors:** Miiri Kotche, Anthony E. Felder, Kimberlee Wilkens, Susan Stirling

**Affiliations:** 1 Richard and Loan Hill Department of Bioengineering, University of Illinois at Chicago, Chicago, IL USA; 2 School of Design, University of Illinois at Chicago, Chicago, IL USA

**Keywords:** Education, Clinical immersion, Bioengineering, Biomedical engineering, Undergraduate, Needs identification

## Abstract

Opportunities to provide clinical immersion experiences to bioengineering undergraduate students have expanded over the last several years. These programs allow students to observe the clinical environment in order to better understand workflow processes, the context in which medical equipment is used, and identify unmet needs firsthand. While each program focuses on identifying unmet needs, these experiences vary in content and implementation. Here we discuss features of clinical immersion programs, share details of our program after six years, and present data regarding post-graduation employment of our participants. Students who participated in the University of Illinois at Chicago Clinical Immersion Program are not more likely to pursue careers in industry as compared to non-participants, nor do they demonstrate an ability to find a job more quickly than non-participants. However, participants who did enter into industry self-reported that the program was impactful to both their career interests and ability to find their first employment position.

## Introduction

Much effort has been focused on ensuring that engineering education reflects contemporary technology development and innovation. In the early 1990s, K-12 education shifted to emphasize the philosophy of “learning by doing”, giving rise to the pedagogical approach of contextual learning.^16^ Contextual learning is based on a constructivist theory of teaching and learning whereby meaning emerges from the relationship between content and its context.^4^,^16^ This approach relates subject matter to real-world situations, thereby allowing students to make connections between knowledge and their own experiences. Thus, as educators were assessing the core competencies and skillsets necessary for engineers in the rapidly changing workplace, contextual learning was emphasized as an integral component of engineering education.^4^ The foundational concepts of engineering and science remain essential, but engineers must also be able to critically assess opportunity and apply knowledge to a variety of problems. Educators, then, should help students learn to bridge boundaries between disciplines and make connections that produce deeper insights.^5^ These findings are reflected in a growing body of recent literature, as well as by the National Academy of Engineering in *The Engineer of 2020: Visions of Engineering in the New Century*^24^ and *Educating the Engineer of 2020: Adapting Engineering Education to the New Century.*^25^ Ongoing discussions regarding integration of “learning by doing” continue to inform curriculum development. In fact, for the field of bioengineering in particular, 300 + educators representing over 100 programs recently gathered to discuss current core competencies for undergraduates at the fourth BME Education Summit.^37^

For bioengineering students, contextual learning can be used to teach the design of medical devices, which requires an understanding of the device, performance requirements, and use in its native environment. Historically, there has been limited, if any, exposure to the clinical environment in the undergraduate bioengineering curricula, and the design of devices and medical equipment has primarily focused on technical functionality. Yet the ability for bioengineers to observe how users in the clinical or hospital environment interact with medical devices enables the informed design of these devices. When bioengineering students work in collaboration with end users and other stakeholders (e.g., clinicians, patients), they’re empowered to identify unmet needs and opportunities for innovation. Thus, clinical immersion programs help develop students’ ability to communicate across discipline boundaries and understand why designs must meet multiple realistic constraints.^21^

In fact, the FDA has increasingly emphasized the importance of identifying user needs and understanding user-device interaction to avoid safety issues, product recalls, and patient harm.^36^ The FDA guidance document *Do It By Design* stresses the value of obtaining first-hand feedback from physicians, nurses and lay-users in the earliest stages of product conception and design.^29^ Extended exposure to the clinical environments helps provide this insight into how behaviors, opinions, and environments inform the user experience. Furthermore, ANSI/AAMI Standard HE75^3^*Human Factors Engineering – Design of Medical Devices,* issued in 2009, focuses on the incorporation of user considerations, including user feedback for iterative design refinements, environmental considerations, anthropometry, and user needs. Literature has also shown that primary ethnographic research, including both observations of and interviews with users in their natural environment, is critical to the successful design of medical devices.^13^,^20^,^22^,^27^,^41^ It is evident that increased attention is being given to “early and often” interaction with end users to enhance the design process of medical devices.

### Clinical Immersion Programs Across the U.S.

To improve undergraduate bioengineering students’ ability to identify and validate true clinical needs and develop solutions according to a user-centered design process, clinical immersion programs have been offered over the last ten years at institutions across the country. Table 1 lists the clinical immersion programs funded through NIH/NIBIB R25 “Team-Based Design in Biomedical Engineering Education”.^2^,^7^,^12^,^15^,^17^–^19^,^23^,^31^,^32^,^34^,^40^ Many of these programs have been informed by the Biodesign Program at Stanford University.^6^ The Biodesign program consists of several components, including a 10-month Innovation Fellowship for participants who have relevant prior experience and/or advanced degrees in STEM, healthcare, business, law, or design,^33^ and courses at both the graduate and undergraduate levels. In 2009, a Biodesign textbook (and later a second edition in 2015) was released, which details the program’s clinical needs identification and development process for medtech innovation.^39^

**Table 1:** Clinical immersion programs for bioengineering undergraduates funded through NIBIB R25 “Team-Based Design in Biomedical Engineering Education”.

Boston University	University of Arizona
Case Western University	University of California—Berkeley
Clemson University	University of California—Davis
Colorado State University	University of California—Los Angeles
Drexel University	University of California—San Diego
North Carolina Agricultural and Technical State University	University of Cincinnati
Northwestern University	University of Delaware
Rowan University	University of Illinois at Chicago
Rutgers University	University of Michigan—Ann Arbor
SUNY—Stony Brook	University of Utah
Temple University	University of Virginia
Tulane University	Wayne State University
Union College	Widener University

Broad guidance from Biodesign and federal funding has helped expand the number of clinical immersion programs offered to bioengineering students. While a needs-based approach is common, content and implementation varies.^1^,^2^,^8^,^12^,^15^,^17^–^19^,^21^,^23^,^28^,^30^,^34^,^38^,^40^ Program content is scoped to leverage institutional strengths and emphasize selected areas of focus such as technology transfer, commercialization, FDA regulatory considerations, insurance reimbursements, or intellectual property. Further, some programs foster interdisciplinary teamwork among engineering disciplines (undergraduate and graduate), business, nursing, and medical students. While programs also differ in duration, ranging from multiple hour-long shadowing sessions to semester- and year-long experiences, most of the literature describe paid, summer-based internships ranging from 5 to 10 weeks. Programs also seek to address the challenge of scalability, for example, by having the clinician available in the classroom as instructors to describe the clinical experience firsthand or by using a team leader model, wherein a student with primary experience diffuses information to the rest of the team.

In this report, we evaluate the impact of the Clinical Immersion Program at the University of Illinois at Chicago on participant career intentions and post-graduate outcomes. We tested the hypothesis that participation in a clinical immersion program would affect post-graduation outcomes compared to non-participants. To the best of our knowledge, this is the first publication to report on post-graduation outcomes of students who participated in a clinical immersion program.

## Materials and Methods

### Development of the Clinical Immersion Program at the University of Illinois at Chicago

The University of Illinois at Chicago (UIC) is a large, public, Research I institution situated in the urban center of the city and is recognized as being among the top 5% most ethnically diverse colleges and universities in the country.^10^ The University of Illinois College of Medicine is one of the largest medical schools in the country and distinguishes itself by its ability to provide medical students with clinical experiences early in their training. The Richard and Loan Hill Department of Bioengineering is uniquely situated to provide a strong clinical immersion experience; in 2011 the Department became jointly operated by the College of Medicine and the College of Engineering. This enables placement of bioengineering students in a wide variety of clinical environments with strong medical faculty support and engagement.

The Clinical Immersion Program (CIP) at UIC was designed to enhance bioengineering student preparedness for industry positions through exposure to clinical-based needs identification as a method to enhance the design of medical devices. This program provides direct exposure to the clinical environment and methodical approaches to observe, interview, identify, and prioritize user needs for effective and efficient engineering design. Over the course of the program, a wide variety of clinical departments have participated in CIP, as listed in Table 2.

**Table 2:** University of Illinois hospital departments/divisions participating in Clinical Immersion Program 2014–2019.

Anesthesiology	Interventional Radiology	Orthopedics
Cardiology	Neurosurgery	Pulmonary Critical Care
Emergency Medicine	Neurology	Radiation Oncology
Gastroenterology	Obstetrics/Gynecology	Transplant Surgery
Hematology/Oncology	Ophthalmology	Urology

CIP was first offered in 2014 as a 6-week, summer-based internship taught by faculty from bioengineering and design. Nine rising senior bioengineering students were selected from a pool of applicants and placed in two, 3-week long clinical rotations. Each week began with a half day of guided instruction in a workshop setting focused on user-centered design, ethnographic research, and opportunities for teams to analyze and synthesize their clinical observations. Students spent the remainder of the week (Tuesday through Friday) in their clinical rotations (at least 30 h/week) under the direction of a physician mentor. Program emphasis was placed on generating meaningful needs statements based on the team observations. Ultimately, teams generated two needs statements, one per clinical rotation. Recognizing the “deep dive” engineering students take into the clinical environments, participants were asked to keep a blog to encourage reflection on their experiences.^9^,^18^

In 2015, the program was expanded to 12 undergraduate bioengineering students as well as additional clinical departments.^18^ We also included Friday workshops for teams to further analyze clinical observations and research solutions to identified needs. In 2016, medical students from the co-curricular Innovation in Medicine program were included in CIP to introduce interdisciplinary teaming.^35^

### Clinical Immersion Program at UIC, 2017–Present

**Figure 1:**
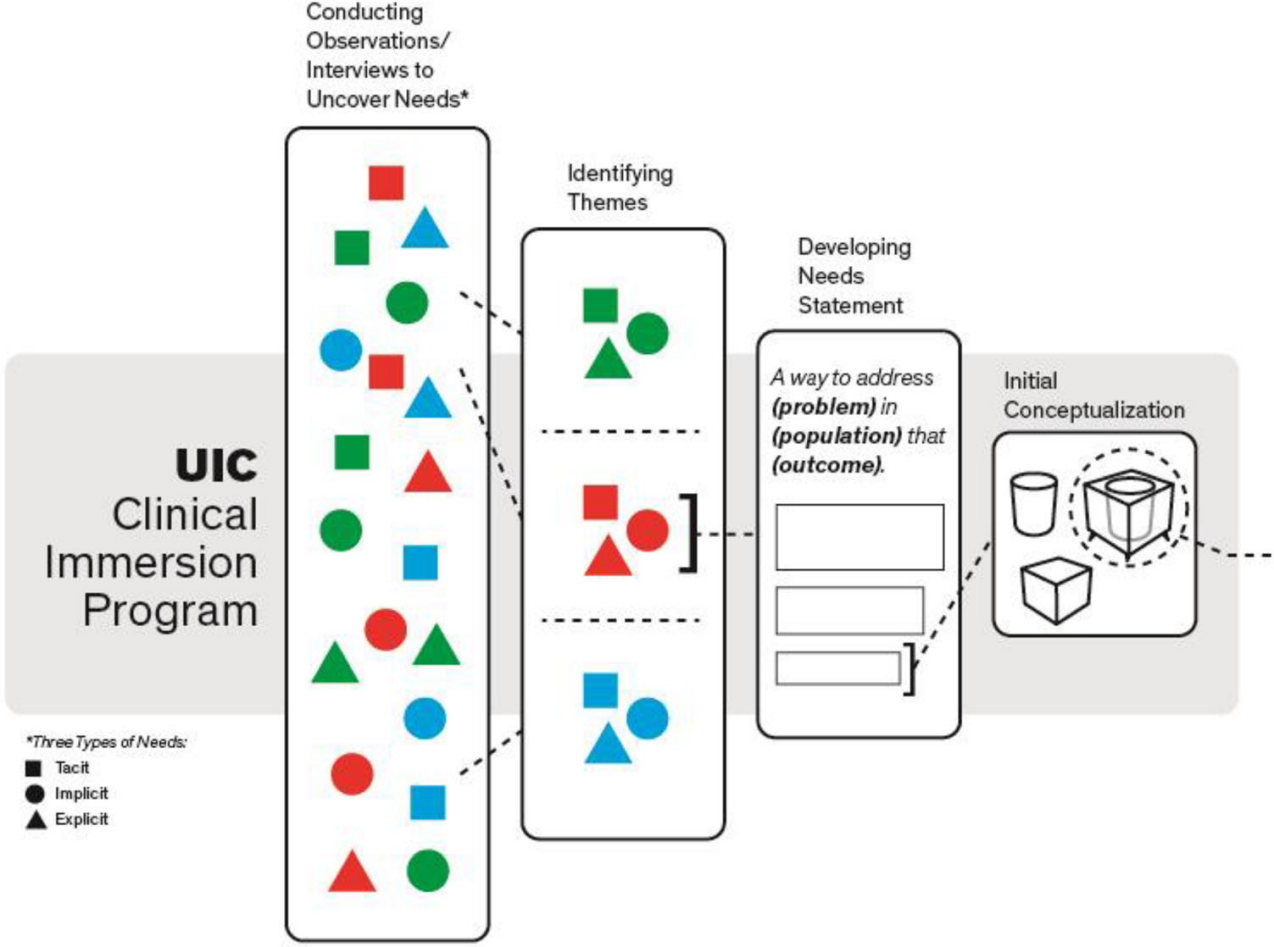
An illustration of the Clinical Immersion Program process at the University of Illinois at Chicago. From Left to Right, raw observations are collected by interdisciplinary student teams in the clinical setting, organized into themes, from which needs statements are developed. The process concludes with initial conceptualization corresponding to the most compelling need statement.

In 2017, the program was modified to allow interdisciplinary teams to spend the full 6-week duration of the program in a single clinical department.^11^ There were several drivers for this change: the desire for students to become more familiar with a clinical specialty, recognize repeated workarounds, identify more meaningful clinical needs, and frame needs identification as a step in the engineering design process instead of a discrete and separate exercise. In addition, this modification was influenced by repeated feedback from participants to explore potential “high-level” solutions in order to better understand the clinical need. While the program expanded to include generating high-level concept solutions, the emphasis remained on identifying and validating unmet user needs. An illustration of our process is provided in Fig. 1 and an outline of weekly topics is provided in Table 3. Briefly, Monday workshops occupied a whole day and consisted of both lectures and activities. Activities reinforced the lecture content in a team setting and included time to practice using tools introduced in lecture, teambuilding and empathy exercises, idea mapping, needs statement development, and concept generation. From Tuesday to Friday, teams spent between 20 and 30 h in their assigned clinical environment as guided by the weekly outline. As part of this process, teams select a high priority unmet clinical need statement and aim to validate it using additional research and initial low fidelity concepting. These concepts, often crafted out of basic art materials, help teams better understand the underlying needs statement by identifying what technical requirements and constraints are relevant to potential solutions.^11^ Further, these concepts facilitate critical discussion with users regarding what constitutes an effective solution. Students found this approach tangible and rewarding, and felt they had a better understanding of the impact when they could explore potential solutions as well as obtain feedback from their clinical mentors. The program instructors actively encouraged both bioengineering and medical students to continue developing solutions for these unmet needs as their capstone projects.

**Table 3:** An outline of the weekly workshops from the Clinical Immersion Program at the University of Illinois at Chicago.

Week	Lecture	Activity/tools
1	Introduction to clinics, CIP, user-centered design, framing a clinical need	Introduction; Guest speakers: surgeon innovator and empathy activity lead; Observations framework for ethnographic observations
2	Interviewing, mapping, and storyboarding	Mapping and categorizing observations, evaluating themes
3	Developing needs statements	Prioritizing themes, developing needs statement
4	Design requirements and criteria, brainstorming	Reflect on needs statements with stakeholders, consider impactful solutions
5	Concept development and iterative prototyping	Ideation on low-fidelity solutions for communication with stakeholders
6	Prep time for final presentations and team reports	
	Final presentations	

### Surveys

This research was approved by an Institutional Review Board at the University of Illinois at Chicago. To assess the effect of CIP on student outcomes, two mixed methods surveys were administered. The first survey (graduation survey) was administered to graduating undergraduate students each spring semester from 2015 to 2018, corresponding to CIP participants from 2014 to 2017. This survey was designed to provide information on students’ experience at UIC and to identify post-graduate career intent. GPA was obtained from college records for comparative analysis. The second survey (follow-up survey) was administered in the spring of 2019 to all undergraduates graduates from 2015 to 2018. This survey was designed to identify student outcomes and related quantifiable metrics. Both surveys were administered using Qualtrics.

### Statistical Analyses

Differences in graduating GPA were determined by unpaired *t* test. Differences in the proportion of students who had advanced placement (AP) college credits or participated in undergraduate research or internship was determined by Fisher’s exact test.

From the graduation survey, the effect of CIP on students’ post-graduation intent (i.e., industry or other) was determined by Fisher’s exact test. The administered survey originally included four options for post-graduation intent (graduate school, professional school, industry, or other). However, the CIP participant cohort was limited in number and stratification into additional groups was precluded. Therefore, post-graduate intent was binarized.

From the follow-up survey, the effect of CIP on graduates’ placement (i.e., industry or other), was determined by Fisher’s exact test. Similar to the graduation survey, these outcomes were binarized from four original options. For the graduates who entered industry, the effect of CIP on time required to finding employment was determined by unpaired *t* test. Differences in the proportion of students whose industry jobs related to engineering was determined by Fisher’s exact test. CIP participants who entered industry were also asked if the program was part of their interview (yes or no), how CIP had impacted their ability to find said position (Likert scale), and how CIP impacted their career interests (Likert scale). Both 5-point Likert scales extended from strongly negative to strongly positive. Statistical analyses were performed using SPSS (Chicago, v.26) and significance was accepted at *p* ≤ 0.05.

## Results

Between 2015 and 2018, 214 undergraduate students graduated from the Richard and Loan Hill Department of Bioengineering from UIC. Of these graduates, 178 (83.1%) completed the graduation survey and 76 (35.5%) completed the follow-up. Between these two surveys, 67 (31.3%) respondents qualified for paired analysis, including 18 who had participated in CIP between 2014 and 2017.

Comparisons between respondents stratified by survey type and participation in CIP are presented in Table 4. Students who participated in CIP had a significantly higher graduating GPA than non-participants (*p* = 0.011). However, CIP participants were not more likely to have AP credits or to have an undergraduate research or internship experience prior to graduating (*p* ≥ 0.116).

**Table 4:** Comparisons between respondents stratified by (1) survey type and (2) participation in the Clinical Immersion Program. Follow-up survey outcomes reflect first placement after graduation.

Survey	Metric	CIP (*N* = 18)	Non-CIP (*N* = 49)	*p* value
Graduation	GPA (4 point scale)	3.48 ± 0.386	3.18 ± 0.469	0.011
	Percentage of students having AP credits	77.8% (14/18)	67.3 (33/49)	0.176
	Percentage of students having undergraduate research experience or internship	88.9% (16/18)	73.4 (36/49)	0.116
	Percentage of students intending on: *Industry*	72.2% (13/18)	65.3 (32/49)	0.2053
	Percentage of students intending on: *Other*	27.8% (5/18)	34.7 (17/49)	0.2053
Follow-up, 1-4 years post-graduation	Percentage of graduates in: *Industry*	72.2% (13/18)	62.3 (31/49)	0.1865
	Percentage of graduates in: *Other*	27.8% (5/18)	36.7 (18/49)	0.1865
	For graduates in Industry: months to employment	2.85 ± 2.3	3.74 ± 4.85	0.411
	For graduates in Industry: Percentage of positions related to engineering	76.9% (10/13)	87.1 (27/31)	0.2348

At the time of graduation, 72% of CIP participants intended to enter industry, compared to 67% of non-participants. Immediately after graduation, 72% of the CIP participants entered into industry (although not the same students who responded affirmatively in the graduation survey), as opposed to 57% of the non-participants. However, CIP participants were not more likely to either intend on or actually enter into industry than non-participants (*p* ≥ 0.1865). Here, ‘other’ career paths include graduate and professional school. From the follow-up survey, two respondents (from non-CIP) did not select between “industry” or “graduate or professional school” as an outcome, and an additional two selected both. The two graduates who selected both industry and schooling were categorized as having gone to industry since that preceded further schooling.

Of the respondents that entered industry immediately after graduation, there was no significant difference in the amount of time to finding that position between the two groups (*p* = 0.411). Nor were CIP participants more likely to find a position related to engineering than non-participants (*p* = 0.2348). However, a majority of the CIP participants in industry (9/13) indicated that the program was part of their job interview. Moreover, all CIP participants indicated that the program had between a positive and strongly positive impact on both getting their position after graduation (3.46 ± 0.78) and their career interests (3.61 ± 0.50).

## Discussion

Much of the published literature on clinical immersion for bioengineering students describes program structure, but there is limited information regarding programmatic outcomes. Program assessments report participants’ ability to understand the patenting, regulatory, and reimbursement processes, ability to identify unmet needs, evaluation of pre/post program career aspirations, engineering design self-efficacy, number of hours spent in the clinic, number of needs identified, number of resulting senior design projects, and understanding of entrepreneurial concepts.^15^,^17^–^19^,^23^,^28^,^30^,^34^,^38^,^40^ However, most of the outcomes rely on participants’ self-reported feedback. While self-reports are valid as a general measure of achievement, self-perceptions of competence may be influenced by factors other than those which are being assessed.^14^ Indirect measures of student performance, including self-perception of student learning, are nevertheless an important component in the holistic assessment of student learning and can provide contextual information about how and why learning is occurring. In this paper, we present both objective metrics and subjective student self-reporting to holistically understand student outcomes.

Students who participated in CIP at UIC had a significantly higher GPA than those who did not participate. This is consistent with the application process for CIP, which considers GPA as one criterion for selection. However, participants in CIP were not more likely to have AP credits or an undergraduate research experience or internship prior to graduation. This suggests that the students participating in CIP were, at least in part, representative of the broader bioengineering student population at UIC.

Students who participated in CIP were not more likely to intend on or ultimately work in industry immediately after graduation compared to non-participants, despite that being the reason the program was initially developed at UIC. Moreover, CIP participants were not more likely to find positions related to engineering, and there was no difference in the time it took for students to obtain their first employment. Collectively, these metrics do not indicate an effect of CIP on student outcomes. However, most CIP participants in industry indicated that CIP was part of their interview, and in general, participants indicated the program was impactful on their career interests.

Some CIP participants provided the following clarifying comments from the follow-up survey:

…doing the clinical immersion program during my time at school made me a competitive candidate for the jobs I had after college.

Clinical Immersion program and [another program] were my favorite parts of my UIC experience and I gained valuable experience from them that helped me get my first job.

These comments suggest that CIP can be an impactful experience for participants. This is consistent with our findings from previous publications.^11^,^18^,^35^ Indeed, this experience may provide a sense of self-efficacy for participants, which has been demonstrated to positively impact student confidence and achievement.^26^ This may be at least partially attributable to the interdisciplinary nature of our program, as one participant reported:

I cannot overstate the importance of the Clinical Immersion Program… to my success post-graduation. Understanding how different disciplines integrate has been the most useful skill for me. This is something that I completely attribute to these two programs.

Our program is dynamic and was among the first of such programs for undergraduate bioengineering students funded by NIBIB in 2014, which has allowed us to reflect on successes and challenges of clinical immersion for bioengineers. We have found increasing success in transitioning projects from CIP to our senior design class. Immersion programs tend to focus on needs identification, and the continuation of the engineering design cycle in a traditional design class is a natural progression. This is especially true for participants of the program, who tend to highly value conceptualization.^11^ Participants in CIP have repeatedly expressed a desire to further understand the clinical need by evaluating how a particular concept solution will address the underlying issue. The transition from identifying clinical needs in CIP to senior design projects has also, at least in part, tried to address scaling and continuity. Immersion programs tend to be able to accommodate a limited number of students due to constraints of the clinical environment, so providing a validated clinical need and dedicated clinical mentor to a senior design team benefits students beyond the summer immersion program. Our program has also evolved to include interdisciplinary collaboration between bioengineering and medical students.^35^ This dynamic has been beneficial, though challenging. Appropriate and realistic expectations must be set and managed for all team members, particularly if there are considerable age and experience gaps among participants. Further, in general, participants of innovation programs benefit from a codified process and methodology,^39^ which we found to assist in setting expectations and potentially circumventing poor team dynamics.

Those interested in establishing their own clinical immersion program would benefit from substantial advanced preparation. The program requires significant administrative support to coordinate hospital clearance (e.g., HIPAA, immunizations), HR clearance (e.g., compensation) as well as scheduling within specific clinics. Further, it is imperative to communicate clear expectations for participating clinical faculty, particularly since this experience differs greatly from a typical clinical shadowing experience. Careful attention must be given to select program applicants, considering professionalism, maturity and self-initiative. Finally, the development of a process that leverages the strengths of one’s institution is critical for a successful program. There is also advantage in ongoing refinement to ensure that the program continues to meet the needs of students.

There were limitations to the current study. First, the limited number of survey respondents, particularly in the CIP participant cohort, makes statistical significance more difficult to demonstrate. Statistical significance in our measured outcome metrics may exist but could not be demonstrated in the current study. Second, the outcome data presented in this study represents the immediate post-graduate effects of CIP. Longer retrospective studies may elucidate other potential differences between cohorts. Third, student outcomes and career placement are the result of complicated and numerous influences. It can be potentially misleading to attribute outcomes to the influence of a single program. However, responses from CIP participants indicate a positive impact of the program regarding their career interests and placement. Fourth, the program has steadily changed since its inception, which can alter our ability to determine an effect of the program on student outcomes. However, the central theme of the Clinical Immersion Program has been and continues to be exposure to the clinical environment and stakeholders in order to better understand their needs. This continued focus provides a consistent experience for participants across all program years.

The Clinical Immersion Program at the University of Illinois at Chicago was designed to enhance bioengineering student preparedness for industry positions by providing direct exposure to the clinical environment and teach methodical approaches to observe, interview, identify, and prioritize user needs for efficient and effective engineering design. Since the program’s inception, it has grown dynamically to accommodate extended exposure in a single clinical department, interdisciplinary teaming, and early concept development. Here we assessed the effects of CIP on student outcomes. To the best of our knowledge, this is the first report to evaluate the impact of a clinical immersion program on student career paths. There was no effect of CIP on the likelihood of students to intend on or enter into industry, as compared to graduate, medical or other professional training, but the participants who did enter into industry indicated the program was impactful to both their career interests and ability to find an industry position. Future work could involve robust evaluation of senior design projects to compare projects stemming from the Clinical Immersion Program and improved post-graduation surveys controlling for multiple variables with increased sample sizes. In addition, it would be beneficial to obtain employer feedback about our graduates to determine any difference between groups.

## References

[1] Allen RH, Acharya S, Jancuk C, Shoukas AA (2013). Sharing best practices in teaching biomedical engineering design. Ann. Biomed. Eng..

[2] Allen, T. E., and D. Chen. Patient centered design in undergraduate biomedical engineering. Paper presented at 2018 ASEE Annual Conference & Exposition, Salt Lake City, Utah, 2018. https://peer.asee.org/30860.

[3] ANSI/AAMI Standard HE75 (2009). Human Factors Engineering—Design of Medical Devices.

[4] Berns RG, Erickson PM (2001). Contextual Teaching and Learning: Preparing Students for the New Economy.

[5] Bordogna J, Fromm E, Ernst EW (1993). Engineering education: innovation through integration. J. Eng. Ed..

[6] Brinton TJ, Kurihara CQ, Camarillo DB, Pietzsch JB, Gorodsky J, Zenios SA, Doshi R, Shen C, Kumar UN, Mairal A, Watkins J, Popp RL, Wang PJ, Makower J, Krummel TM, Yock PG (2013). Outcomes from a postgraduate biomedical technology innovation training program: the first 12 years of Stanford Biodesign. Ann. Biomed. Eng..

[7] Cash, H. L., J. D. DesJardins, and B. Przestrzelski. The DMVP (detect, measure, valuate, propose) method for evaluating identified needs during a clinical and technology transfer immersion program. Paper presented at 2018 ASEE Annual Conference & Exposition, Salt Lake City, Utah, 2018. https://peer.asee.org/31087.

[8] Choi, J. H. Work in progress: The incorporation of hands-on, team-based design challenges in a large enrollment introductory biomedical engineering course. Paper presented at 2016 ASEE Annual Conference & Exposition, New Orleans, Louisiana, 2016. https://peer.asee.org/27042.

[9] Clinical Immersion Program at the University of Illinois at Chicago. https://clinicalimmersion.uic.edu. Accessed 5 Feb 2020.

[10] College Factual: UIC Scores High Marks for Ethnic Diversity. https://www.collegefactual.com/colleges/university-of-illinois-at-chicago/news/university-of-illinois-at-chicago-2019-college-diversity-ethnic-ranking/. Accessed 6 Feb 2020.

[11] Felder, A. E., M. Kotche, S. Stirling, and K. M. Wilkens. Interdisciplinary clinical immersion: from needs identification to concept generation. Paper presented at 2018 ASEE Annual Conference & Exposition, Salt Lake City, Utah, 2018. https://peer.asee.org/30699.

[12] Freeborn, T., and M. Gosa. Board 13: work in progress: pilot shadowing experiences to introduce engineering students to speech-language pathology. Paper presented at 2018 ASEE Annual Conference & Exposition, Salt Lake City, Utah, 2018. https://peer.asee.org/29918.

[13] Gertner, M. Biomedical innovation, surgical innovation, and beyond. In: Proceedings from the National Collegiate Inventors and Innovators Alliance, p. 277, 2005.

[14] Gonyea RM (2005). Self-reported data in institutional research: review and recommendations. New Dir. Inst. Res..

[15] Guilford, W. H., M. Keeley, B. P. Helmke, and T.E. Allen. Work in progress: a clinical immersion program for broad curricular impact. Paper presented at 2019 ASEE Annual Conference & Exposition, Tampa, Florida, 2019. https://peer.asee.org/33581. 10.18260/1-2--33581 PMC8479758 34594158

[16] Johnson EB (2002). Contextual Teaching and Learning: What It is and Why It’s Here to Stay.

[17] Kadlowec, J., T. Merrill, S. Sood, J. Greene Ryan, A. Attaluri, and R. A. Hirsh. Clinical immersion and team-based design: into a third year. Paper presented at 2017 ASEE Annual Conference & Exposition, Columbus, OH, 2017. https://peer.asee.org/28040.

[18] Kotche M. Clinical Immersion Internship Introduces Students to Needs Assessment. Paper presented at 2016 ASEE Annual Conference & Exposition. New Orleans, Louisiana, June 2016. https://peer.asee.org/26503.

[19] Logsdon, E. A., R. Allen, N. J. Durr, and H Nguyen. Board # 11: a team leader model for biomedical engineering design team project-definition training and scalable clinical observation (work in progress). Paper presented at 2017 ASEE Annual Conference & Exposition, Columbus, OH, 2017. https://peer.asee.org/27687.

[20] Maguire M (2001). Methods to support human-centred design. Int. J. Hum. Comput. Stud..

[21] Mittal V, Thompson M, Altman SM, Taylor P, Summers A, Goodwin K, Louie AY (2013). Clinical needs finding: developing the virtual experience—a case study. Ann. Biomed. Eng..

[22] Money AG, Barnett J, Kuljis J, Craven MP, Martin JL, Young T (2011). The role of the user within the medical device design and development process: medical device manufacturers’ perspectives. BMC Med. Inform. Decis. Mak..

[23] Muller-Borer, B. J., and S. M. George. Designing an interprofessional educational undergraduate clinical experience. Paper presented at 2018 ASEE Annual Conference & Exposition, Salt Lake City, Utah, 2018. https://peer.asee.org/30279.

[24] National Academy of Engineering (2004). The Engineer of 2020: Visions of Engineering in the New Century.

[25] National Academy of Engineering (2005). Educating the Engineer of 2020: Adapting Engineering Education to the New Century.

[26] Pajares F, Schunk DH (2001). Self-beliefs and school success: self-efficacy, self-concept, and school achievement. Perception.

[27] Privitera, M. B., and D. L. Murray. Applied ergonomics: determining user needs in medical device design. In: 2009 Annual International Conference of the IEEE Engineering in Medicine and Biology Society, pp. 5606–5608, 2009. 10.1109/IEMBS.2009.5333781 19964396

[28] Przestrzelski, B., J. D. DesJardins, and C. M. I. Brewer. Year two—The DeFINE program: A clinical and technology transfer immersion program for biomedical needs identification and valuation. Paper presented at 2016 ASEE Annual Conference & Exposition, New Orleans, Louisiana, 2016. https://peer.asee.org/27062.

[29] Sawyer D (1996). An introduction to Human Factors in Medical Devices.

[30] Schmedlen, R., J. W. Lee, P. Shekhar, and J. Stegemann. The clinical peer mentors program: student motivations, skills and knowledge acquisition, and influence on career path. Paper presented at 2019 ASEE Annual Conference & Exposition, Tampa, FL, 2019. https://peer.asee.org/33376.

[31] Singh A, Ferry D, Balasubramanian S (2019). Efficacy of clinical simulation-based training in biomedical engineering education. J. Biomech. Eng..

[32] Sood, S., M. Short, and R. Hirsh. Biodesign through clinical immersion. In: Proceedings from the National Collegiate Inventors and Innovators Alliance, 2015.

[33] Stanford Biodesign Innovation Fellowship. http://biodesign.stanford.edu/programs/fellowships/innovation-fellowships/program-details.html. Accessed 6 Feb 2020.

[34] Stephens, J. S., S. I. Rooney, E. S. Arch, and J. Higginson. Bridging courses: unmet clinical needs to capstone design (work in progress). Paper presented at 2016 ASEE Annual Conference & Exposition, New Orleans, Louisiana, 2016. https://peer.asee.org/26393.

[35] Stirling, S., and M. Kotche. Clinical immersion program for bioengineering and medical students. Paper presented at 2017 ASEE Annual Conference & Exposition, Columbus, OH, 2017. https://peer.asee.org/28041.

[36] U.S. Food and Drug Administration. Human factors and medical devices. http://www.fda.gov/medical-devices/device-advice-comprehensive-regulatory-assistance/human-factors-and-medical-devices. Accessed 6 Feb 2020.

[37] White JA, Gaver DP, Butera RJ (2020). Core competencies for undergraduates in bioengineering and biomedical engineering: findings, consequences, and recommendations. Ann. Biomed. Eng..

[38] Yazdi Y, Acharya S (2013). A new model for graduate education and innovation in medical technology. Ann. Biomed. Eng..

[39] Yock PG, Zenios S, Makower J, Brinton TJ, Kumar UN, Watkins FT, Denend L, Krummel TM, Kurihara CQ (2015). Biodesign: The Process of Innovating Medical Technologies.

[40] Zapanta, C. M., H. D. Edington, P. E. Empey, D. C. Whitcom, and A. J. Rosenbloom. Board # 18: clinical immersion in a classroom setting (work in progress). Paper presented at 2017 ASEE Annual Conference & Exposition, Columbus, Ohio, 2017. https://peer.asee.org/27799.

[41] Zoltowski CB, Oakes WC, Cardella ME (2012). Students’ ways of experiencing human-centered design. J. Eng. Ed..

